# Evaluating the Accuracy of Molecular Diagnostic Testing for Canine Visceral Leishmaniasis Using Latent Class Analysis

**DOI:** 10.1371/journal.pone.0103635

**Published:** 2014-07-30

**Authors:** Manuela da Silva Solcà, Leila Andrade Bastos, Carlos Eduardo Sampaio Guedes, Marcelo Bordoni, Lairton Souza Borja, Daniela Farias Larangeira, Pétala Gardênia da Silva Estrela Tuy, Leila Denise Alves Ferreira Amorim, Eliane Gomes Nascimento, Geraldo Gileno de Sá Oliveira, Washington Luis Conrado dos-Santos, Deborah Bittencourt Mothé Fraga, Patrícia Sampaio Tavares Veras

**Affiliations:** 1 Laboratório de Patologia e Biointervenção, Centro de Pesquisa Gonçalo Moniz–Fundação Oswaldo Cruz, Salvador, Bahia, Brazil; 2 Escola de Medicina Veterinária, Universidade Federal da Bahia, Salvador, Bahia, Brazil; 3 Instituto de Matemática –Departamento de Estatística, Universidade Federal da Bahia, Salvador, Bahia, Brazil; 4 Centro de Referência em Doenças Endêmicas Pirajá da Silva (PIEJ), Jequié, Bahia, Brazil; 5 Instituto Nacional de Ciência e Tecnologia em Doenças Tropicais (INCT - DT), Salvador, Bahia, Brazil; Instituto Oswaldo Cruz, Fiocruz, Brazil

## Abstract

Host tissues affected by *Leishmania infantum* have differing degrees of parasitism. Previously, the use of different biological tissues to detect *L. infantum* DNA in dogs has provided variable results. The present study was conducted to evaluate the accuracy of molecular diagnostic testing (qPCR) in dogs from an endemic area for canine visceral leishmaniasis (CVL) by determining which tissue type provided the highest rate of parasite DNA detection. Fifty-one symptomatic dogs were tested for CVL using serological, parasitological and molecular methods. Latent class analysis (LCA) was performed for accuracy evaluation of these methods. qPCR detected parasite DNA in 100% of these animals from at least one of the following tissues: splenic and bone marrow aspirates, lymph node and skin fragments, blood and conjunctival swabs. Using latent variable as gold standard, the qPCR achieved a sensitivity of 95.8% (CI 90.4–100) in splenic aspirate; 79.2% (CI 68–90.3) in lymph nodes; 77.3% (CI 64.5–90.1) in skin; 75% (CI 63.1–86.9) in blood; 50% (CI 30–70) in bone marrow; 37.5% (CI 24.2–50.8) in left-eye; and 29.2% (CI 16.7–41.6) in right-eye conjunctival swabs. The accuracy of qPCR using splenic aspirates was further evaluated in a random larger sample (n = 800), collected from dogs during a prevalence study. The specificity achieved by qPCR was 76.7% (CI 73.7–79.6) for splenic aspirates obtained from the greater sample. The sensitivity accomplished by this technique was 95% (CI 93.5–96.5) that was higher than those obtained for the other diagnostic tests and was similar to that observed in the smaller sampling study. This confirms that the splenic aspirate is the most effective type of tissue for detecting *L. infantum* infection. Additionally, we demonstrated that LCA could be used to generate a suitable gold standard for comparative CVL testing.

## Introduction

Visceral leishmaniasis (VL) is a disease with both medical and veterinary importance that is endemic in Brazil, and in many other countries throughout Latin America, Asia, and Europe [1]. One of the etiological agents of VL is *Leishmania infantum* (syn. *Leishmania chagasi*), which is transmitted to vertebrate hosts through the bites of female sand flies [2]–[5].

Dogs are considered the main domestic reservoir for this parasite because of their high rates of infection and the high frequency of parasites found in their skin [6]–[9]. Once infected with *L. infantum*, dogs have clinical manifestations that range from asymptomatic to systemic, including weight loss or cachexia; hypertrophy of the lymph nodes; and changes to the skin such as onychogryphosis, footpad swelling, localized or generalized alopecia, skin ulcers, and nasal or periocular dermatitis. They can also present with pathological alterations such as anemia or hepatic and renal failure [10], [11].

Canine visceral leishmaniasis (CVL) can be diagnosed using parasitological, serological, or molecular methods in conjunction with clinical and epidemiological parameters [12]. Serological tests to diagnose CVL are the most common procedures used worldwide [13], however they lack sensitivity and specificity, which makes diagnosing the disease difficult when animals present with low antibody titers or there is cross-reactivity [14]–[17]. Hence, additional tests could be advantageous for confirming the diagnosis of inconclusive cases. For use as a confirmatory test, the molecular detection of *Leishmania* spp. provides greater sensitivity and specificity than other diagnostic techniques [8], [18].

Numerous studies have described highly sensitive detection of low parasitic loads using quantitative real-time PCR (qPCR) [19]–[21]. qPCR has also been used to monitor the tissue parasitic load in dogs following anti-*Leishmania* treatment in countries where this procedure is unrestricted [22], [23].

Several invasive, and non-invasive, techniques have been used to obtain biological tissue samples to diagnose *Leishmania* infection using conventional PCR and qPCR. The biological samples most widely used for molecular diagnosis of *Leishmania* spp. infection in dogs are the spleen, bone marrow, lymph node, and skin [12], [18], [24]. However, molecular diagnostic tests in studies using these tissue types have produced variable, and sometimes conflicting results, for identifying *Leishmania*-infected dogs [19], [25], [26]. This might be because culturing the parasite, which has been used as the gold standard assay [27], [28], has a low sensitivity threshold for detecting dogs with a low parasite burden [29], [30], which compromises the accuracy evaluation of diagnostic testing.

Therefore, the authors hypothesized that the lack of a reliable gold standard assay could account for the varying accuracy of the molecular diagnostic tests for *Leishmania* infection in different tissues. Latent class analysis (LCA) appraises tests with imperfect reference standards [31]–[33] using a statistical model to construct the latent class variable. Recently, LCA has been used to accurately evaluate the results of serological tests for diagnosing CVL [34].

The aim of the present study was to determine which type of canine tissue sample in an area with endemic VL provided the highest rate of *Leishmania* DNA detection by qPCR. In addition, qPCR results were compared to parasitological and serological diagnostic tests to determine which test provided the most accurate diagnosis of *L. infantum* infection.

## Materials and Methods

### 1. Ethics Statement

Experimental procedures involving dogs were performed in accordance with Brazilian Federal Law on Animal Experimentation (Law no. 11794), the guidelines for animal research established by the Oswaldo Cruz Foundation [35], and the Brazilian Ministry of Health Manual for the Surveillance and Control of VL [36]. The CPqGM - FIOCRUZ Institutional Review Board for Animal Experimentation approved protocols for both animal euthanasia and sample collection procedures (Permit Number: 015/2009; Permit Number 017/2010).

### 2. Dogs

As previously described by Lima et al. (2014), over a one week period in July 2010, 51 stray dogs were taken from the streets of Jequié, a municipality located in the State of Bahia, Brazil, which is an area endemic for CVL. These dogs were selected as part of a surveillance and control program for VL that our group conducted in collaboration with the Endemic Diseases Surveillance Program of the State Health Service [37]. A CVL diagnosis was established based on the presence or absence of the following clinical signs: emaciation, alopecia, anemia, conjunctivitis, dehydration, dermatitis, erosion, ulcerations, lymphadenopathy, and onychogryphosis as previously detailed by Lima et al. (2014). Dogs from Jequié were clinically classified as having mild (stage I), moderate (stage II), and severe CVL (stage III) according to Solano-Gallego et al. (2009) [38].

### 3. Tissue Sampling

Tissue samples were obtained during necropsies as previously described by Lima et al. (2014). Briefly, the dogs were anesthetized and then euthanized by intracardiac injection of a supersaturated solution of potassium chloride (2 mL/kg). Immediately before the lethal injection, 50 mL of blood were collected by intracardiac puncture. Blood samples were preserved in EDTA-2Na tubes (Greiner bio-one, Kremsmünster, Austria) and in blood collection tubes (BD Vacutainer; Becton, Dickinson and Co). During the necropsy, splenic aspirate samples were collected by puncturing the central region of the spleen and bone marrow samples were obtained by puncturing the wing of the ilium, approaching from the dorsal crest. Conjunctival swabs of the right and left eyes were taken by rubbing the swab multiple times against the surface of the lower eyelid. A small fragment of the popliteal lymph node was cut from the whole organ and a skin fragment was collected using a sterile 5 mm punch (Kolplast, Brazil) from the medial portion of the pinna. Tissue samples were collected using sterile needles, swabs, and blades and all of the samples were stored in DNAase- and RNAase-free tubes at −70°C until DNA extraction.

### 4. Hematological and Biochemical Parameters

Hematological and biochemical parameters were evaluated on the day of the necropsy. Total red blood cell and white blood cell counts were determined using an automated cell counter (Pentra 80 counter, ABX Diagnostics, Montpellier, France). Micro-hematocrit tubes containing blood samples were centrifuged at 12,000 rpm for 5 min, and then the hematocrit levels were estimated. Serum was collected by centrifuging the Vacutainer tubes, and was used for the biochemical tests including total protein, globulin, albumin, blood urea nitrogen, and creatinine, using an enzymatic colorimetric method with an A15 auto-analyzer (BioSystems, Barcelona, Spain).

### 5. Serological and Parasitological Tests

The following serological tests were performed to detect anti-*Leishmania* antibodies: the DPP CVL rapid test which detects rk28-specific antibodies and the EIE CVL with crude *L. major* antigen diagnostic test provided by FIOCRUZ (Bio-Manguinhos Unit, Rio de Janeiro, Brazil). These serum tests were performed in accordance with manufacturer instructions. An in-house ELISA, with crude *L. infantum* antigen was also performed as previously described [39], [40]. Parasitological evaluation was performed by culturing part of the splenic aspirate collected during necropsy in Novy–MacNeal–Nicolle (NNN) biphasic medium supplemented with 20% Fetal Bovine Serum (FBS – Gibco BRL, New York, USA) and 100 µg/mL gentamicin to avoid contamination (Sigma Chemical Co., St. Louis, MO) for four weeks at 24°C [41]. Parasites were detected using microscopy performed at weekly intervals for no less than four weeks. Each splenic culture was prepared in duplicate. All of the culture labels were double-checked to avoid misidentification.

Parasite isolates were randomly selected from five dogs and sent to the national reference laboratory for *Leishmania* typing at the Oswaldo Cruz Institute (CLIOC, Rio de Janeiro, RJ, Brazil). The isolates were typed using monoclonal antibodies and enzyme electrophoresis analysis in order to determine the *Leishmania* species.

### 6. Control Samples

Splenic aspirate samples from 20 dogs that had previously been identified as *Leishmania-*positive from an endemic area [18] were used as positive controls. Splenic aspirates of 20 healthy dogs from the municipality of Pelotas, Rio Grande do Sul, Brazil, an area without endemic CVL, were used as negative controls. All of the healthy dogs had no clinical signs of CVL, and tested negative for infection using the in-house ELISA, parasite culturing, and qPCR techniques.

### 7. Sample Handling and Decontamination Procedures

Due to the high degree of sensitivity inherent in qPCR, exceptional care was taken to avoid cross-contamination during not only the sample collection procedures, but also during DNA extraction and qPCR testing. As previously described [18], all procedures were carried out in an environment that was suitable for sample collection and qPCR procedures. All of the disposable surgical materials were used for a single animal, and the laminar flow hood was decontaminated by UV radiation before each procedure. Filter tips were routinely used throughout all DNA extraction steps and when performing the qPCR [42].

### 8. DNA Extraction

DNA was obtained from 200 µL of splenic and bone marrow aspirate, 200 µL of blood, 20 mg of lymph node, and 20 mg of a skin fragment using a DNeasy Blood & Tissue Kit (Qiagen, Hilden, Germany) in accordance with the manufacturer’s protocols. DNA samples from the conjunctival swabs were purified using a phenol–chloroform method as previously described [42]. The DNA pellets were suspended in 30 µL of Tris–EDTA buffer (10 mmol/L Tris and 1 mmol/L EDTA, pH 8.0). Once extracted, the quality and concentration of each DNA sample were evaluated using a digital spectrophotometer (NanoDrop ND-1000, Thermo Scientific, Wilmington, USA) [43]. All of the DNA samples were adjusted to a final concentration of 30 ng/µL, aliquoted, and kept at −20°C until the qPCR assays were performed.

Parasite DNA was extracted from *L. infantum* (MHOM/BR2000/MERIVALDO), *Leishmania amazonensis* (MHOM/Br88/Ba-125), *Leishmania braziliensis* (MHOM/BR/94/H3456), and *Leishmania major* (MHOM/RI//WR-173) promastigotes cultivated at 24°C. For the DNA extraction, the parasites were counted and centrifuged. DNA was extracted from pellets corresponding to a known number of parasites in accordance with the Qiagen protocols.

### 9. Quantitative PCR (qPCR)

#### 9.1 Inclusion and exclusion criteria

To assess positivity, DNA samples were only included in the analysis if they met the minimum quality criteria: i) the DNA sample concentration was above 30ng/µl; ii) DNA samples amplified with the same efficiency as the DNA curve; and iii) amplification of the 18s rRNA housekeeping gene was successful. Any samples that did not fulfill one or more of the above inclusion criteria were excluded, only 10 out of 51 for skin fragments and 26 out of 51 for bone marrow aspirate. To compare parasitic load in different tissue types, DNA samples were only included in the analysis if they met the minimum quality criteria for all tissue types (samples from 20 dogs out of 51).

#### 9.2 Quantitative PCR Assay

qPCR was used to determine the amount of parasite DNA in canine tissue samples. qPCR assays were performed following an amplification protocol previously described by Francino et al. (2006). The qPCR technique targeted a conserved region of *L. infantum* kDNA to obtain a 120-bp amplicon. All of the reactions were performed in triplicate. The reaction was in a final volume of 25 µL containing: 5 µL (150 ng) of each DNA sample diluted in deionized water and 20 µL of the PCR mixture. The PCR mixture contained: 12.5 µL of Universal Mastermix (Life Technology Corporation, Carlsbad, CA-USA), the forward primer 5′-AACTTTTCTGGTCCTCCGGGTAG-3′ (LEISH-1) and the reverse primer 5′-ACCCCCAGTTTCCCGCC-3′ (LEISH-2) both at a final concentration of 900 nM, and a fluorogenic probe 5′-AAAAATGGGTGCAGAAAT-3′ with a FAM reporter molecule attached to the 5′ end and an MGB-NFQ quencher (200 nM final concentration) linked to the 3′-end (Life Technology Corporation). In order to overcome limitations caused by endogenous PCR inhibitors in the blood, skin fragment, and conjunctival swab samples, all of the steps leading up to DNA amplification were performed in the presence of bovine serum albumin (5 µg/each reaction) (Sigma Chemical) [44].

#### 9.3 Quantification of *Leishmania* kDNA

Quantification of *Leishmania* kDNA was performed using an absolute method based on comparing the cycle threshold (Ct) values from the samples to a standard curve, which was constructed using serial 10-fold dilutions from 10^5^ to 10^−1^ parasites performed in triplicate. Reactions were performed using the Applied Biosystems 7500 Fast Real-Time PCR System (Life Technology Corporation). The reaction was carried out under the following conditions: 1 cycle at 50°C for 2 min, 1 cycle at 95°C for 10 min, and 40 two-step cycles, first at 95°C for 15 s and then at 60°C for 1 min. In order to minimize variability between plates, the values from each plate were normalized using a common fluorescence detection baseline. Each sample’s Ct value was calculated by determining the point at which its fluorescence signal was above the established detection baseline. The Ct cut-off value was determined using a Receiver-Operator Characteristic (ROC) curve. The optimal Ct cut-off value for the parasite kDNA qPCR assay was determined by calculating sensitivity and specificity for different Ct cut-off points and the ROC curve derived from the amplification values of *Leishmania*-negative samples and *Leishmania-*positive samples (see item 6). Tissue samples were considered positive when the Ct values were equal to or less than the Ct cut-off point determined using the ROC curve analysis. If the standard deviation between triplicates was >0.38, the sample set was reanalyzed by qPCR [45]. The efficiency of the qPCR protocol was evaluated by calculating the slope value of the standard curve for the parasite kDNA. This value, −3.657 (SD = 0.148), was obtained from the mean slope values of nine independent experiments with a correlation coefficient (R^2^) of 0.998.

#### 9.4 Assessment of qPCR Analytical Sensitivity and Specificity

Analytical sensitivity was evaluated by determining whether the presence of host tissue interferes with the amplification profiles when using qPCR to detect *L. infantum* DNA in infected dogs. First, a standard curve was constructed using ten-fold dilutions from reference strain *L. infantum* DNA (see item 9.3). Next, a ten-fold dilutions of reference strain *L. infantum* DNA was mixed with the splenic aspirate DNA from negative control animals (see item 6) and another standard curve was constructed from these dilutions. Finally, the amplification profiles of the two curves were compared. The analytical specificity of the qPCR analysis was assessed by comparing the amplification profiles of DNA samples from the *L. infantum* reference strain to profiles from several other *Leishmania* species, including the New World *L. amazonensis* and *L. braziliensis*, and the Old World *L. major*. As described in item 9.3, standard curves for each species were constructed from ten-fold serial dilutions ranging from 10^5^ to 10^−1^ parasites performed in triplicate. Analytical specificity was further assessed by evaluating the amplification profiles of DNA obtained from other canine pathogens, such as *Ehrlichia canis* and *Babesia canis*. Briefly, 150 ng of DNA from each pathogen was amplified and compared to the *L. infantum* amplification profile.

#### 9.5 Quantification of 18S rRNA Gene Expression

The expression of the canine housekeeping gene 18S rRNA was measured in order to normalize the concentration of input DNA for each sample and to obtain a reference amplification value to ensure the use of high-quality DNA samples [46]. TaqMan Pre-Developed Assay Reagents (Life Technology Corporation) were used to detect and quantify 18S rRNA gene expression. All of the reactions were performed at a final volume of 25 µL containing: 5 µL of DNA canine tissue sample diluted in deionized water and 20 µL of PCR mixture. The PCR mixture contained: 12.5 µL of Universal Mastermix (Life Technology Corporation), 1.25 µL of 18S GeneEx Assay primer and probe sets (Life Technology Corporation) at a concentration of 20x, and deionized water to obtain the final volume. The positive and negative controls for the housekeeping genes were plated in triplicate and the samples were plated in duplicate. Reactions were performed on an Applied Biosystems 7500 Fast Real-Time PCR System (Life Technology Corporation) using the following protocol: 1 cycle at 50°C for 2 min; 1 cycle at 95°C for 10 min; and 40 two-step cycles, first at 95°C for 15 s and then 50°C for 1 min. A seven point standard curve was constructed for the housekeeping gene ranging from 450–18.75 ng. The slope of the standard curve for the 18s rRNA gene was −3.399 (SD = 0.296), which represents the mean slope value of 11 independent experiments with the corresponding coefficient of determination (R^2^) of 0.990.

#### 9.6 Parasitic Load in DNA Samples

Samples from 20 of the 51 dogs were used to determine which tissue type harbored the highest parasitic load by comparing the splenic and bone marrow aspirates, blood, conjunctival swab of right and left eyes, lymph node and skin fragments. The parasitic load was expressed as the number of parasites normalized to the established reference amplification value for the 18S rRNA gene in 150 ng of DNA from each tissue sample [47]. Then the value obtained was calculated per 100 mg of host tissue DNA.

### 10. Evaluation of qPCR accuracy using splenic aspirate samples from a prevalence study

The accuracy of the qPCR assay was evaluated using splenic samples obtained from 800 dogs during a random prevalence study performed in Camaçari, BA, an endemic area for CVL in Brazil. All 800 dogs were clinically evaluated and classified as described in item 2. They were also tested using the following CVL diagnostic methods: DPP CVL rapid test, EIE CVL, our in-house ELISA, and parasite cultures from splenic aspirates as described in item 5. qPCR analysis of splenic aspirate samples was performed as described in item 9.

### 11. Statistical Analysis

In order to prevent bias, serological, parasitological and molecular techniques were performed and their results were judged without knowledge of the outcome of the other tests.

The ROC curve data analysis described in item 9.3 was performed using GraphPad Prism software v.5.0 (GraphPad Prism Inc., San Diego, CA). Differences in the parasitic load between each type of biological sample were assessed using the Friedman test followed by the Dunn’s multiple comparison test. The relationship between parasitic load in the spleen and qPCR positivity in each infected tissue was assessed with the Spearman correlation test using log transformed values for the parasitic load (*p*<0.05).

For the 800 dogs evaluated in the cross sectional study, the intensity of the parasitic load in the spleen (item 9.6) was categorized into three ranges: <10^4^, 10^4^–10^6^, and >10^6^. The number of clinical signs in the dogs (item 2) was stratified into four ranges: 0 (no clinical signs), 1–3, 4–6, and >6 clinical signs. Fisher’s exact test was used to evaluate the association between the number of clinical signs and the splenic parasitic load ranges.

LCA was performed using a statistical model to define a latent variable that could be used as a gold standard. To define a latent variable that could accurately identify *L. infantum* infection, three indicators representing serologic (DPP CVL), parasitological (culture from splenic samples), and molecular (splenic aspirate qPCR) diagnostic techniques were included. Animals were grouped into two categories, ‘infected dogs’, and ‘not-infected dogs’. The latent classes were estimated and characterized using two parameters: (a) item-response probabilities and (b) class prevalence, which is the probability of belonging to a latent class according to the response pattern. The estimate was performed using the maximum likelihood with expectation-maximization (EM) algorithm. The goodness of fit of the statistical model was evaluated using entropy, which varied between 0 and 1, with the value 1 indicating that the individuals are perfectly classified into the latent classes. Average probabilities for each latent class, which expresses the uncertainty of global classification, were also assessed *a posteriori,* considering a higher *a posteriori* probability to be a better goodness of fit for the statistical model. The Vuong-Lo-Mendell-Rubin likelihood ratio test was used to choose the number of classes in LCA [48]. The Akaike information criterion (AIC) and Bayes information criterion (BIC) were also evaluated for each model. LCA was performed using the software Mplus 5.2, the syntax for fitting LCA in MPlus program is reported in Appendix S1 [49]. Additionally, the conditional independence was checked by evaluation of significant bivariate residuals [50], [51].

The sensitivity and 95% confidence interval (CI) were calculated for each diagnostic technique and each tissue type analyzed, using the LCA latent variable as gold standard. The accuracy (sensitivity and specificity) of the qPCR technique using splenic aspirates was further evaluated with the LCA in a random sample of 800 dogs. Sensitivity of each test was measured as the proportion of positive results, only among those identified as such by the gold standard, while specificity was measured as the proportion of negative results, which were correctly identified as such by the gold standard.

## Results

### 1. Sample description

All 51 dogs from the endemic area of Jequié were mixed-breed, their estimated ages varied from 1–10 years old, the animals weighed 5–30 kg, 45% (23/51) were males, and 55% (28/51) were females. All of the dogs exhibited clinical signs that could be related to CVL including splenomegaly (33/51), emaciation (17/51), hypertrophy of the lymph nodes (46/51), alopecia (21/51), cutaneous alterations (41/51), onychogryphosis (29/51), and ocular alterations (10/51). With respect to clinical pathology, 73% of the dogs presented with anemia (35/48), 98% with hypergammaglobulinemia (49/50), and 98% with hypoalbuminemia (49/50). Using the scale published by Solano-Gallego et al. (2009), all of the dogs were classified as having moderate CVL (stage II), except one animal that also exhibited a creatinine value greater than 1.4 mg/dL and was considered to have severe CVL (stage III).

### 2. Standardization of the qPCR Protocol

The Ct cut-off value for parasite DNA detection was performed using a ROC analysis. This analysis showed an area under the curve of 1.0, indicating a high probability (*p*<0.001) that a randomly chosen positive sample would be correctly classified. The Ct cut-off value of 37.0 had prediction rates of 100% sensitivity (CI 83.16–100) and 95% specificity (CI 75.13–99.87) with a likelihood ratio of 20. The analytical sensitivity was then determined. We found that the amplification profile of the reference strain *L. infantum* DNA was similar to that of the reference strain mixed with splenic aspirate DNA from negative control animals. The lower limit of detection was then determined and corresponded to 0.016 parasites per reaction.

In terms of the analytical specificity, the Old World *L. major* parasite DNA samples were remarkably similar to those of *L. infantum* at all of the concentrations tested. In contrast, DNA from *L. amazonensis* and *L. braziliensis* could only be successfully amplified at concentrations of 10^4^ and 10^5^ parasites per reaction. This corresponded to the same number of cycles needed to amplify DNA from 0.02 parasites per reaction of the *L. infantum* reference strain (Figure S1). *E. canis* and *B. canis* DNA did not amplify using this qPCR protocol (data not shown). With respect to the housekeeping gene, attempts to amplify18S rRNA from DNA samples of *Leishmania* spp. resulted in no detectable qPCR amplification using the same primer set that successfully amplified the gene in canine DNA samples (data not shown).

### 3. Positivity of diagnostic techniques

Using qPCR, 100% of the dogs from Jequié (51/51) tested positive for parasite DNA in at least one of the tissue types analyzed. Among these, 98% (50/51) tested positive in the splenic aspirate samples; 80.4% (41/51) in blood samples; 68.3% (28/41) in skin fragments; 54.9% (28/51) in lymph node fragments; 35% (7/20) in bone marrow aspirate; 37.3% (19/51) in left eye conjunctival swabs, and 33.3% (17/51) in right eye conjunctival swabs.

Parasites were observed in 35.3% (18/51) of the parasite cultures from splenic aspirate and anti-*Leishmania* antibodies were detected in 43.8% (21/48), 47.1% (24/51), and 66.7% (34/51) of the canine serum samples using the EIE CVL, DPP CVL rapid test, and in-house ELISA, respectively.

### 4. Accuracy of the diagnostic tests

Latent class was used to provide a reliable estimate of sensitivity and specificity in order to select the tissue that provided the greatest accuracy for qPCR DNA detection. Serological, parasitological, and molecular techniques were used to determine prevalence of the latent classes and conditional probabilities in the LCA model for *L. infantum* infection in dogs. The probability that a dog from Jequié would be classified as infected using the LCA model was 47.1%. Among the animals considered infected by the LCA, the probability that a dog would test positive using qPCR of the splenic aspirate was 95.8%. The probability that a dog tested positive using either DPP CVL or by parasite culture from splenic aspirates was 100.0% or 54.2%, respectively (Table 1).

**Table 1:** Prevalence of latent classes and conditional probabilities to the LCA model for *L. infantum* infection detection in dogs.

		Dogs from Jequié n = 51			Dogs from Camaçari n = 800		
Technique	Result		Latent Classes			Latent Classes	
			Infectedn = 24 (47.1%)	Not Infectedn = 27 (52.9%)		Infectedn = 120 (14.5%)	Not Infectedn = 680 (85.5%)
		Result Frequency (%)	Conditional Probabilities (%)		Result Frequency (%)	Conditional Probabilities (%)	
**DPP CVL**	**Positive**	47.1	100.0	0.0	16.6	82.9	5.5
	**Negative**	52.9	0.0	100.0	83.4	17.1	94.5
**Splenic Aspirate Culturing**	**Positive**	35.3	54.2	18.5	13.2	87.8	0.0
	**Negative**	64.7	45.8	81.5	86.8	12.2	100.0
**Splenic Aspirate qPCR**	**Positive**	98.0	95.8	100.0	34.2	93.3	24.1
	**Negative**	2.0	4.2	0.0	65.8	6.7	75.9

Entropy was then calculated to assess how well the animals were classified *a posteriori* by the model. The entropy of the Jequié samples was 1.0; indicating accuracy in the classification of dogs using LCA. Moreover, *a posteriori* average probabilities that animals were properly classified into the latent classes “Infected” and “Not Infected” were 100% in both cases in the Jequié animals. The Lo-Mendel-Rubin test indicated that the model with 2 classes was a better fit for the data obtained from the Jequié dogs (*p*<0.01) when compared with the model with only 1 class (data not shown). These results are supported by the analysis of the AIC and BIC (data not shown).

**Figure 1:**
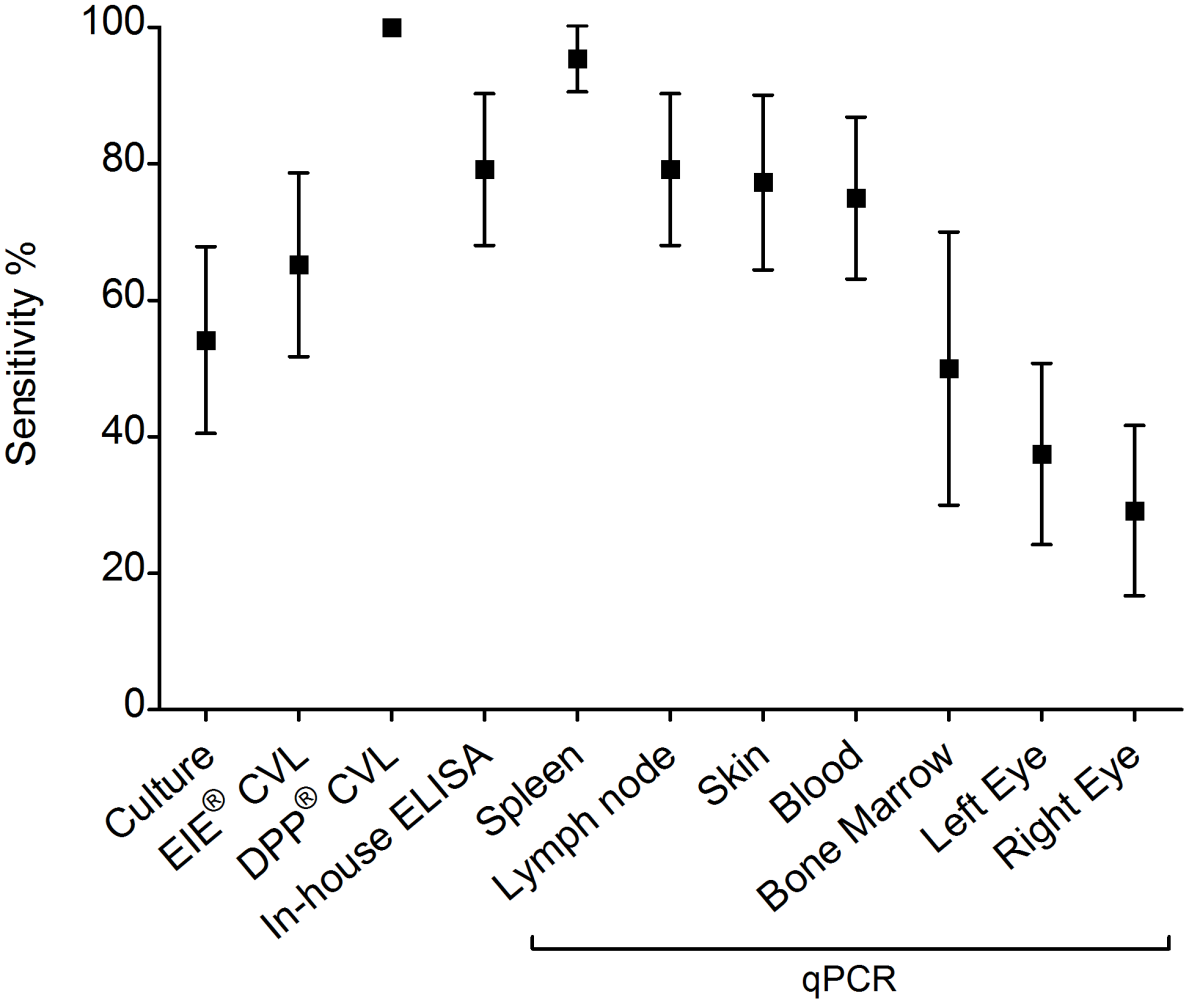
Sensitivity of the different diagnostic techniques employed in the biological samples obtained from Jequié animals (n = 51). Vertical bars represent the 95% confidence intervals. Sensitivity values were obtained using the latent variable as the gold standard.

The sensitivity of the tests employed in Jequié to diagnose *L. infantum* infection was assessed employing the latent variable obtained by LCA as the gold standard (Figure 1). Splenic aspirates provided the highest sensitivity of the available tissues sampled achieving 95.8% (95%CI 90.4–100) of sensitivity. The sensitivity attained in other tissues ranged from 80% to 30% as follows: lymph node fragments 79.2% (95%CI 68–90.3), skin fragments 77.3% (95%CI 64.5–90.1), blood 75% (95%CI 63.1–86.9), bone marrow aspirates 50% (95%CI 30–70), left eye swab 37.5% (95%CI 24.2–50.8), and right eye swab 29.2% (95%CI 16.7–41.6). It was not possible to calculate splenic qPCR specificity since only one sample tested negative in this method. Specificity of the other tissues achieved 66.7% for lymph node fragments (95%CI 53.7–79.6) as well as for bone marrow aspirates (95%CI 47.8–85.6), 63% (95%CI 49.7–76.2) for right and left eye swabs, 42.1% (95%CI 27–57.2) for skin fragments and 14.8% (95%CI 5.1–24.6) for blood. Considering the other diagnostic tests, the sensitivity of the serological tests was 100% for the DPP CVL, followed by 79.2% (95%CI 68–90.3) for the in-house ELISA, 65.2% (95%CI 51.7–78.7) for EIE CVL, while sensitivity for the splenic aspirate culturing was 54.2% (95%CI 40.5–67.8). The specificity was highest for DPP CVL 100%, followed by splenic parasite cultures 81.5% (95%CI 70.8–92.1), EIE CVL 76% (95%CI 63.9–88.1), in-house ELISA 44.4% (95%CI 30.8–58.1).

### 5. Parasitic load in different tissue types

To further characterize tissue performance for the molecular diagnostic assay, parasitic loads were determined in the different tissues analyzed. As shown in Table 2 a considerable degree of variation was observed among the samples with values ranging from 120 parasites in a splenic aspirate sample up to 186 million parasites found in a bone marrow aspirate sample. However, the median parasitic load was higher in splenic aspirate samples than in the conjunctival swabs from either eye (*p*<0.05) or bone marrow aspirate (*p*<0.05). No statistically significant differences were observed when comparing parasitic loads in the splenic aspirate to the blood or skin tissue samples.

**Table 2:** Parasitic loads detected in different canine tissue types from a total of 20 dogs from the endemic area of Jequié.

Tissue type	Positivity	Parasitic loads^a^				
		Minimum	25% Percentile	Median	75% Percentile	Maximum
**Splenic Aspirate**	100% (20/20)	120	1,088	4,365	14,325	74,000,000
**Blood**	70% (14/20)	0	0	7,960	19,800	228,000
**Skin Fragment**	60% (12/20)	0	0	1,870	21,500	32,400,000
**Lymph node Fragment**	60% (12/20)	0	0	830.5	9,288	7,800,000
**Bone Marrow Aspirate**	35% (07/20)	0	0	0.0*	28,275	186,000,000
**Left Eye Swab**	50% (10/20)	0	0	645.0*	2,073	240,000
**Right Eye Swab**	35% (07/20)	0	0	0.0*	3,141	147,000

a number of parasites normalized by the established reference amplification value for the housekeeping gene 18S rRNA in 100 mg of host tissue DNA.

*p<0.05 Friedman’s together with Dunn’s multiple comparisons test of splenic aspirates and swab of right or left eye and splenic aspirates and bone marrow.

### 6. Distribution of parasitic load according to number of clinical signs

The distribution of parasitic load according to the number of clinical signs is displayed in Table 3. We observed a significant positive association between the intensity of parasitic load in the spleen and the number of clinical signs present in the dogs. Animals with no clinical signs (*p*<0.01) or those exhibiting 1–3 clinical signs (*p*<0.001) had lower parasitic loads in splenic tissue (<10^4^). In contrast, animals with >6 clinical signs (*p*<0.01) showed relatively higher loads (>10^6^). The dogs presenting with 4–6 clinical signs were homogeneously distributed throughout the three ranges.

**Table 3:** Distribution of parasitic load according to number of clinical signs in dogs from the prevalence study.

Number of Clinical Signs	Splenic Parasitic Load Ranges			Fisher Exact Test
	<10^4^	10^4^–10^6^	>10^6^	
**0**	8 (57.1%)	5 (35.7%)	1 (7.1%)	*p*<0.01
**1–3**	55 (42%)	49 (37.4%)	27 (20.6%)	*p*<0.001
**4–6**	37 (39.4%)	27 (28.7%)	30 (31.9%)	*p* = 0.11
**>6**	5 (16.1%)	9 (29.0%)	17 (54.8%)	*p*<0.01
**Total**	105	90	75	

### 7. Accuracy of qPCR using splenic aspirate samples from a prevalence study

Splenic aspirate samples collected from a random study conducted in the endemic area of Camaçari were used to evaluate the high sensitivity observed for the qPCR technique developed using convenience sampling from Jequié. Positive diagnoses in the samples from Camaçari varied according to diagnostic test. In this sample, 34.2% were positive using qPCR, 24.4% using EIE CVL, 19.8% using the in-house ELISA, and 16.6% using DPP CVL.

Similar to the samples from Jequié, LCA was used to analyze the results from the Camaçari samples. Reliability of the LCA model was evaluated and the probability of an animal being infected with *L. infantum* was calculated. The response patterns obtained from the latent class model that were used are listed in Table 4. Animals from Camaçari that had at least two positive test results were classified by the LCA model as ‘Infected’. However, the presence of a positive result from the splenic aspirate parasite culture implied a 100% probability of being infected with *L. infantum*, regardless of the DPP CVL and splenic aspirate qPCR results. When dogs from this endemic area tested negative by all three diagnostic techniques, the probability that the animal was infected with *L. infantum* was 0%. Furthermore, the probability of animals being infected was still very low when only splenic aspirate qPCR (2.7%) or DPP CVL (1.4%) tested positive according to this LCA model.

**Table 4:** Response patterns^a^ of Camaçari dogs for LCA model with 2 latent classes for diagnosis of CVL.

Response pattern					
DPP CVL	Splenic AspirateCulturing	Splenic AspirateqPCR	Frequency Observed% (n)	CVL Probability *a posteriori*(%)	Result Basedon LCA
N	N	N	60.1 (429)	0.0	Not infected
N	N	P	20.5 (146)	1.4	Not infected
P	N	N	3.6 (26)	2.7	Not infected
N	P	N	0.1 (01)	100.0*	Infected
P	N	P	2.7 (19)	54.7	Infected
N	P	P	2.1 (15)	100.0	Infected
P	P	N	0.7 (05)	100.0	Infected
P	P	P	10.2 (73)	100.0	Infected

a Response patterns of all samples tested using the three techniques.

*Estimation based on only one animal sample presenting this pattern.

N: Negative; P: Positive.

The entropy of the Camaçari samples was 0.934, and the *a posteriori* average probabilities of being correctly classified as “Infected” and “Not Infected” were, respectively, 92.4% and 99.3%. Similar to the analysis performed with samples from Jequié, using random samples, the Lo-Mendel-Rubin test indicated that the model with 2 classes was optimal and was supported by the analysis of the AIC and BIC (data not shown).

**Figure 2:**
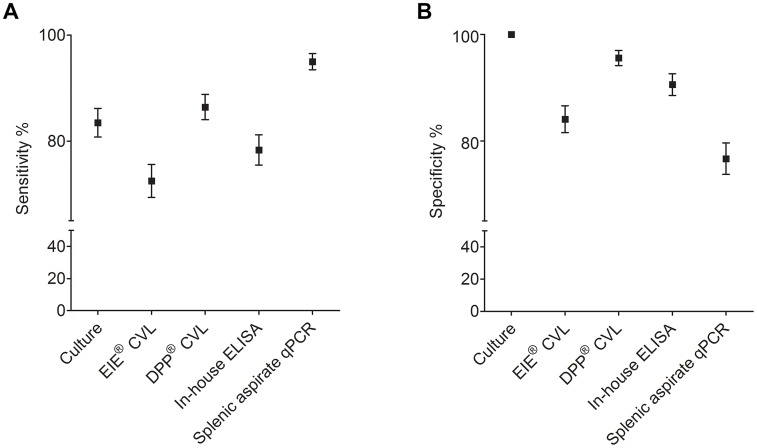
Sensitivity and specificity of the different diagnostic techniques employed in the biological samples obtained from Camaçari animals (n = 800). Vertical bars represent the 95% confidence intervals. **A)** Sensitivity and **B)** Specificity values obtained using the latent variable as the gold standard.

Using LCA, the sensitivity of the splenic aspirate qPCR (95%; 95%CI 93.5–96.5) was higher than for the other diagnostic tests: DPP CVL (86.4%; 95%CI 84.1–88.8), splenic parasite cultures (83.5%; 95%CI 80.8–86.2), the in-house ELISA (78.3%; 95%CI 75.5–81.2), and EIE CVL (72.5%; 95% CI 69.4–75.6) (Figure 2A). However, the specificity was highest for splenic parasite cultures (100%), followed by DPP CVL (95.6%; 95%CI 94.2–97), the in-house ELISA (90.6%; 95%CI 88.6–92.6), EIE CVL (84.1%; 95%CI 81.6–86.6), and splenic aspirate qPCR (76.7%; 95%CI 73.7–79.6) (Figure 2B).

## Discussion

The present study found that a qPCR protocol targeting *Leishmania* kDNA provided the highest diagnostic sensitivity in dogs from Jequié when compared to standard serological and parasitological methods. In this endemic area, the DPP CVL rapid test and EIE CVL were able to detect infection in 47.1% and 43.8%, respectively, of a population of symptomatic dogs. Interestingly, 100% of these dogs tested positive with respect to at least one of the tissue types analyzed using qPCR. Similar results have been obtained by other studies, in which high sensitivity was achieved using molecular techniques [14], [16], [52]. Together these results reinforce the notion that the number of infected dogs detected by serological surveys in endemic areas is severely underestimated [53], [54].

Several methods have been recently developed for the molecular detection of *Leishmania* spp. [20], [21], [55], that provide divergent results when used in a variety of clinical canine samples [54]. Among the tissues analyzed, the authors observed that splenic aspirate samples provided the highest detection rate, successfully identifying 98% of the samples that tested positive. This result is supported by the fact that the spleen is a key site for parasite multiplication in naturally infected dogs [24], [56]. Interestingly, following splenic aspirate samples, 80.4% of blood samples tested positive using qPCR. In addition, we found that the parasitic loads achieved were similar in the blood and splenic aspirate samples. These are promising results given that drawing blood is a much less invasive sampling technique to detect *Leishmania* infection in dogs than obtaining splenic aspirates. In contrast, several other studies have found that bone marrow and lymph node tissues offered a higher number of positive results than blood [46], [55], [57], [58]. Francino et al. (2006) suggested that using qPCR to detect *Leishmania* parasites in blood samples might be sufficient to diagnose infection given the technique’s ability to quantify extremely low parasitemia. However, other authors consider the blood to be a poor source of *Leishmania* DNA [59], mostly because blood samples do not have satisfactory detection rates using conventional PCR. The underlying cause of these poor results may be the high frequency of PCR inhibitors found in blood, in addition to low parasitic loads, which could lead to false negatives especially in asymptomatic dogs [52]. Serum albumin can be added to avoid any potential inhibiting effects in qPCR reaction [44]. In the present study we added serum albumin to blood, skin, and conjunctival swab samples. Our results demonstrate that splenic aspirates or blood can be effectively used to detect parasite DNA using qPCR [18], [19].

The analytical specificity of the qPCR technique was also evaluated in the present study by comparing the amplification profiles of *L. infantum* DNA to other Old and New World *Leishmania* species. The amplification profile of the Old World species *L. major* was remarkably similar to that of *L. infantum* (Figure S1). This corroborates other studies that have shown a great deal of similarity between the genomes of these species [60]. To the best of our knowledge, *L. major* is not known to be a causative agent of CVL, nor have any cases linked to this parasite been reported in Latin America [61]. kDNA from New World parasites, such as *L. amazonensis* and *L. braziliensis*, was successfully amplified using this protocol, but only at high concentrations of 10^4^ and 10^5^ parasites per reaction (Figure S1). Protocols capable of distinguishing between *Leishmania* species are preferable in endemics areas for both cutaneous and visceral forms of the disease [62]. In this study, five *Leishmania* species isolated from the dogs were identified by multilocus enzyme electrophoresis as *L. infantum*. Nonetheless, the use of splenic aspirate samples can avoid misleading diagnostic results since visceralization of *L. braziliensis* has not been reported and visceralization of *L. amazonensis* is a relatively rare event both in humans or dogs [62]–[64].

Regrettably, an ideal gold standard is still lacking for CVL diagnosis [65]. Historically, parasite culturing and immunofluorescence antibody test (IFAT) have been abundantly used. However, culturing is shown to have low sensitivity, while IFAT low specificity [65]. An alternative to using a single technique as the gold standard is to utilize LCA, once this method defines a latent variable to be used as gold standard, considering all diagnostic tests impartially. Indeed, LCA has been proved to successfully estimate the sensitivities and specificities of different diagnostic tests for several diseases [34], [66]–[69]. LCA has been an useful tool for validating serological diagnostic methods for VL, since this analysis provides more realistic estimates of diagnostic test performance [34], [67]. In the scientific community still exist concerns regarding the high sensitivity of qPCR results, especially when this technique is able to detect very low parasitic loads. In addition, some authors state that is impossible for qPCR to differentiate between the DNA of a living parasite and a dead one. Otherwise, Prina et al. (2007) [70] were the only ones that proved that as soon as 1 h after exposure to a substance able to kill the parasites, only less than 1% of the initial *Leishmania* DNA could be detected by qPCR. No other group demonstrated these results, especially using invivo experiments. Thus, in the present study, we have decided not to consider all the dogs as infected, even if they displayed parasite in at least one tissue by the qPCR, and perform the qPCR accuracy evaluation using the latent variable.

Employing the latent class variable as the gold standard, we found that the sensitivity for splenic aspirate qPCR and DPP CVL were 95.8% and 100% respectively, in a population of symptomatic dogs in Jequié. However, these results were limited since it was a small sample size. To address this, the results of the qPCR testing were evaluated using a larger random sampling of dogs that consisted of a population of positive and negative dogs, which are representative of the population of an endemic area for VL. In this random population survey using 800 dogs, the high sensitivity of splenic aspirate qPCR was confirmed achieving 95% of sensitivity, while the DPP CVL sensitivity was corrected to 83.5%. Despite the high sensitivity of the splenic aspirate qPCR, the specificity was relatively low (76.7%). This could be due to the large number of dogs from the randomly sampled population that tested positive only by splenic aspirate qPCR (20.5%) and were considered as ‘Not infected’ by the LCA. These animals were likely misclassified by LCA as false negatives, since the splenic aspirate qPCR is known to be the most sensitive diagnostic technique for CVL, most likely more sensitive than the variables used to define the variable latent class.

Several studies have demonstrated a positive correlation between clinical manifestations of CVL and parasitic load in the spleen, lymph nodes and skin using several techniques [20], [41], [56], [71]. Using qPCR of splenic aspirate in dogs, we also found a positive association between parasitic load and clinical manifestations of CVL, reinforcing the notion that can be used not only for detection of infection but also to monitor disease severity in dogs.

Although splenic aspirate collection is considered an invasive procedure by many dog owners [27], [72], Barrouin-Melo et al. (2006) noted that minor complications were observed in only three out of 257 dogs that underwent splenic aspiration. Complications can be further minimized by visualizing the spleen using an ultrasound device to guide splenic aspiration [72], [73]. In our experience, during the prevalence study in the municipality of Camaçari, the splenic aspirate procedure assisted by ultrasonography was well tolerated in all 800 dogs without any reported complication.

In conclusion, the authors found that, the splenic aspirates and blood, provided the greatest sensitivity for detecting *Leishmania* DNA using qPCR. In addition, the results indicated that LCA could be used to create a suitable gold standard for diagnosis, since this technique offers a more comprehensive evaluation of the results obtained using different diagnostic testing methods for CVL.

## Supporting Information

Figure S1 **Amplification profiles of DNA samples from** ***Leishmania*** **spp. A)** *L. infantum*; **B)** *L. major*; **C)** *L. amazonensis*; **D)** *L. braziliensis*. DNA samples derived from the *L. infantum* reference strain, and several other *Leishmania* species, including New World *L. amazonensis* and *L. braziliensis*, and Old World *L. major*. Standard curves were constructed using amplification patterns from ten-fold serial dilutions performed in triplicate ranging from 10^5^ to 10^−1^ parasites per reaction.
(TIF)

Appendix S1 **Syntax for fitting LCA in MPlus program.**
(DOCX)
