# Supplementary figures and images for: Evaluating the Accuracy of Molecular Diagnostic Testing for Canine Visceral Leishmaniasis Using Latent Class Analysis
Source: PLoS One. 2014 Jul 30;9(7):e103635. doi: 10.1371/journal.pone.0103635 (PMC4116254; doi:10.1371/journal.pone.0103635)

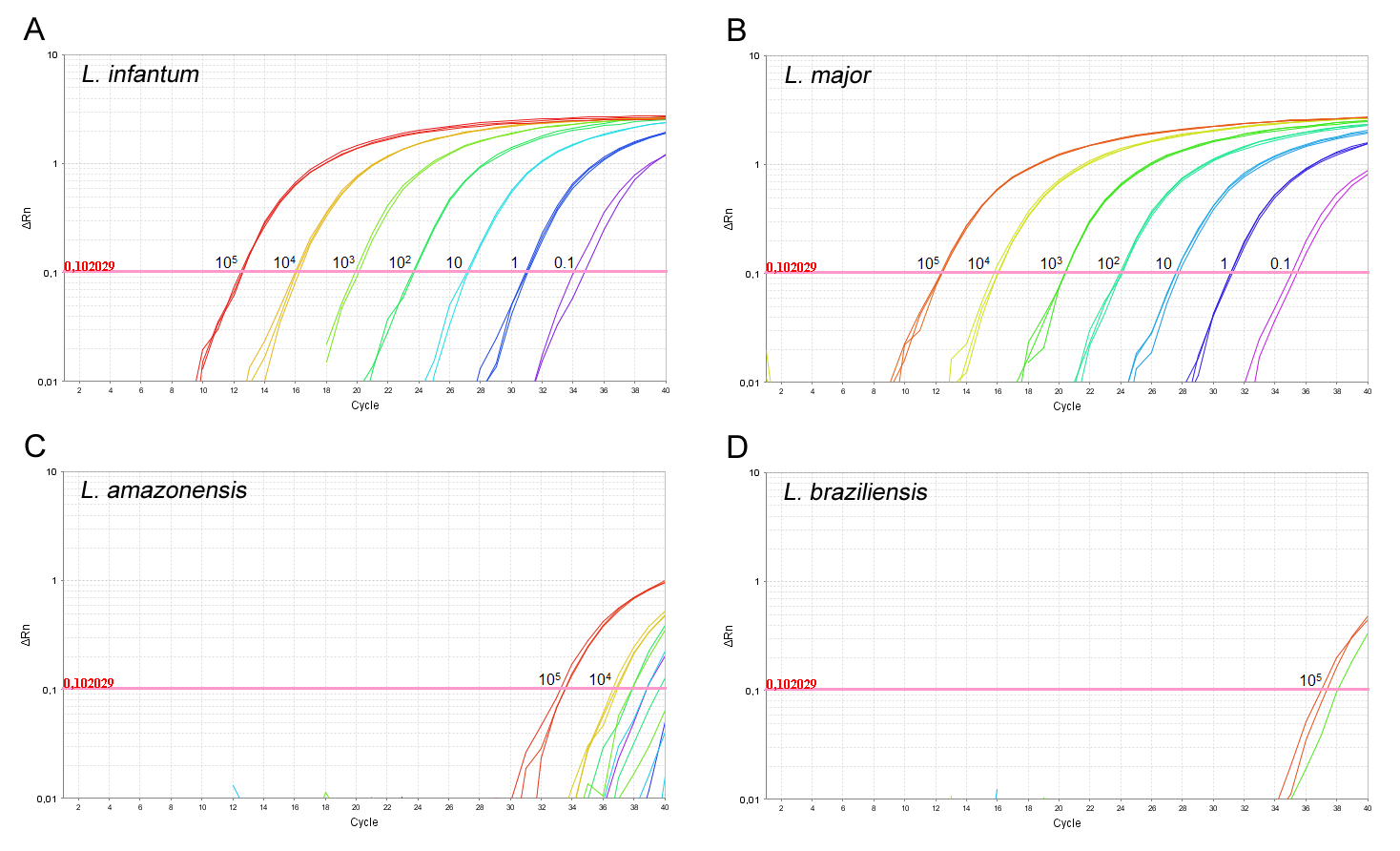

Supplement: Figure S1 — Amplification profiles of DNA samples from Leishmania spp. A) L. infantum; B) L. major; C) L. amazonensis; D) L. braziliensis. DNA samples derived from the L. infantum reference strain, and several other Leishmania species, including New World L. amazonensis and L. braziliensis, and Old World L. major. Standard curves were constructed using amplification patterns from ten-fold serial dilutions performed in triplicate ranging from 105 to 10−1 parasites per reaction. (TIF) [file pone.0103635.s001.tif]
