# Supplementary material for: Evaluating the Accuracy of Molecular Diagnostic Testing for Canine Visceral Leishmaniasis Using Latent Class Analysis
Source: PLoS One. 2014 Jul 30;9(7):e103635. doi: 10.1371/journal.pone.0103635 (PMC4116254; doi:10.1371/journal.pone.0103635)
Supplement: Appendix S1 — Syntax for fitting LCA in MPlus program. (DOCX) [file pone.0103635.s002.docx]

APPENDIX

Mplus Commands for LCA

TITLE: LCA with binary latent class indicators

DATA: FILE IS "dogs.ascii";

VARIABLE:

NAMES ARE id DPPCVL EIECVL ELISA culture spleen blood limph righte lefte skin bonemar;

USEVARIABLES = DPPCVL culture spleen;

CATEGORICAL= DPPCVL culture spleen;

CLASSES = c (2);

MISSING = .;

AUXILIARY = id;

ANALYSIS:

TYPE = MIXTURE;

STARTS = 100 10;

STITERATIONS = 20;

OUTPUT:

TECH11 TECH14 TECH10;

PLOT:

TYPE IS plot3;

SERIES IS DPPCVL (1) culture (2) spleen (3);

SAVEDATA:

FILE IS LCA_dogs.txt;

SAVE IS cprob;

FORMAT IS free;
